# ELISA-Based Crossmatching Allowing the Detection of Emerging Donor-Specific Anti-HLA Antibodies through the Use of Stored Donors' Cell Lysates

**DOI:** 10.1155/2015/763157

**Published:** 2015-11-08

**Authors:** G. Schlaf, K. Stöhr, A. Rothhoff, W. Altermann

**Affiliations:** Tissue Typing Laboratory (GHATT), University Hospital Halle/Saale, Magdeburger Strasse 16, 06112 Halle, Germany

## Abstract

About forty years ago the complement-dependent crossmatch assay (CDC-CM) was developed as standard procedure in order to select recipients without donor-specific antibodies directed against human leukocyte antigens of their given donors since the negative outcome of pretransplant crossmatching represents one of the most important requirements for a successful kidney graft survival. However, as a functional assay the CDC-CM strongly depends on the availability of donors' isolated lymphocytes and in particular on their vitality highly limiting its applicability for recipients treated with special drugs and therapeutic antibodies or suffering from underlying autoimmune diseases. In the great majority of these cases ELISA-based crossmatching has been demonstrated to be an adequate alternative procedure nevertheless leading to valid results. With these case reports we show for the first time that ELISA-based crossmatching is suitable to demonstrate the upcoming donor-specific anti-HLA antibodies as a consequence of allografting using deep-frozen deceased donor's material such as blood or spleen detergent lysate. Thus, this ELISA-based procedure first provides the option to routinely perform crossmatching using stored material of deceased donors in order to substitute or at least to complement virtual crossmatching, that is, the comparison of the recipients' anti-HLA antibody specificities with the donors' historically identified HLA types.

## 1. Introduction

More than 40 years ago the correlation between antibodies which are directed against antigens of donor tissues and hyperacute rejections of allografts was described for the first time [1]. Later studies provided evidence that these donor-specific antibodies (DSA) were in nearly all cases of their detection directed against human major histocompatibility (MHC) antigens, the so-called human leukocyte antigens (HLA) [2, 3]. In order to prevent recipients from hyperacute and acute rejections, the procedure of the complement-dependent cytotoxicity (CDC) assay was developed and established as standard crossmatch (CM) technique in the late sixties of the last century. With regard to the procedure lymphocytes isolated from a given donor's blood are incubated with the prospective recipient's serum to lead to a complement-dependent attack in the presence of added rabbit complement. The outcome is analyzed by calculating the number of dead cells (positive reaction) using two-color fluorescence microscopy. Ethidium bromide as a lethal dye stains only dead cells after their attack by complement components initially activated by bound DSA via the classical pathway of complement activation. Due to technical difficulties the older procedure, that is, the single staining method by means of eosin, is currently used only by a low minority of the tissue typing laboratories. However, using the one or the other staining protocol as a functional assay the CDC generally detects only those antibodies which exert their allogeneic detrimental function by an activation of the complement system. This technique, however, does not identify DSA which lack complement-activating features although these may also be involved in acute rejection episodes and may consequently be detrimental for grafted organs or tissues [4, 5]. Additionally, the CDC is characterized by a low sensitivity which led to its modification named anti-human globulin- (AHG-) enhanced CDC. Secondary anti-human immunoglobulin antibodies directed against the primary DSA are additionally used in order to increase the level of complement activation [6, 7]. Regarding the interpretability of the outcomes, however, all variants of the CDC-CM depend on a high quality of the donor cells and often do not lead to clear results if a given donors' lymphocytes exhibit vitality rates lower than 90%. The same holds true for cell samples contaminated by other leukocytes or precursor cells since the staining procedure leads to interpretable results only with lymphocytes. As an alternative to circumvent some of these CDC-CM-specific problems the procedure of flow cytometric (FACS) crossmatching was first published in 1983 by Garovoy and coworkers [8] leading to the detection of both complement-activating and complement-independent DSA. Although this procedure is characterized by a higher sensitivity which is in the range of the AHG-enhanced CDC [9, 10] it is frequently influenced by false positive outcomes resulting from the “irrelevant” binding of IgG antibodies via their Fc parts to Fc receptors, which are strongly expressed on B-lymphocytes [11, 12]. Consequently, a method has been proposed of performing the B-cell FACS-CM by implementing the use of heat-denatured rabbit serum, highly reducing the background caused by nonspecific IgG binding through their Fc parts [13]. This procedure, already well known for immunohistochemical applications to block Fc*γ* receptors, may first have the capacity to reliably overcome the problem of unspecific binding of antibodies through their Fc parts as this method does not include the disadvantage of an unspecific digest of surface proteins. Former attempts to selectively remove Fc receptors through the use of the enzyme pronase were in many cases not successful most probably due to different activities of the enzyme used. The second striking disadvantage in complete analogy with the CDC-CM is that both assays do not lead to valid results if only cells of poor quality/vitality are available. This drawback led to the generation of procedures which are completely independent of the cell vitality. In this context two assays in the design of solid-phase enzyme based assays (ELISA) were developed in the past: (i) the Antibody Monitoring System (AMS) HLA class I/II ELISA (GTI Diagnostics, Waukesha, USA) and (ii) the AbCross HLA class I/II ELISA (Bio-Rad Medical Diagnostics, Dreieich, Germany). Due to its first commercial availability the AMS-ELISA had already been established in our laboratory in 2005 and, after its discontinuation by the manufacturer for commercial reasons in 2013, was replaced by the AbCross-ELISA in a highly modified manner. Thus, the AMS-ELISA was the first procedure which exhibited complete independence of the quality of the donors' cells. Several publications dealing with the superiority of these solid-phase-based assays over the classical CDC-CM have hitherto been published especially in the context of various factors falsifying the outcome of the CDC-CM. These factors comprise the use of pharmaceutical treatment such as cytostatic agents or therapeutic antibodies [14–17] and accompanying autoimmune diseases [4, 11, 12, 16, 18–20] of a given recipient. Furthermore, the procedure of ELISA-based crossmatching was successfully implemented using the outer corneal rim, that is, highly acellular tissue as the only material available from given donors instead of vital blood lymphocytes [11, 21]. With our cases we first report that this ELISA-based procedure allows repeated runs of crossmatching by using aliquots of the same stored cell lysate of a given donor. Consequently this procedure first offers the possibility to monitor donor-specific antibodies against a graft by* de facto* crossmatching instead of virtual crossmatching, that is, the comparison of the recipient's antibody specificities with the HLA phenotypes of the corresponding donor.

## 2. Case Presentations

### 2.1. Case 1: Confirmation of Donor-Specific Anti-HLA Class I (Anti-HLA-B7) Antibodies by ELISA-Based Crossmatching as Cause for a 42-Year-Old Lung Transplant Recipient's Rejection Episode

**Figure 1:**
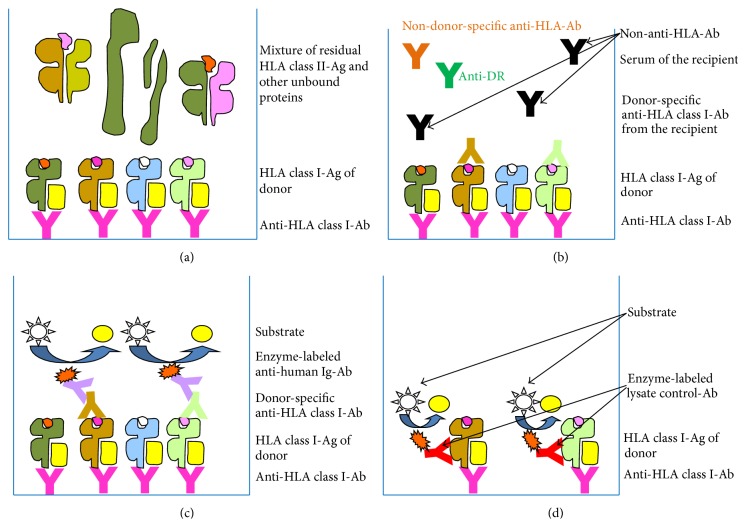
Flow diagram of the AMS-ELISA shown for the detection of HLA class I molecules. (a) Binding of the donor's solubilized HLA class I molecules by monoclonal capture antibodies recognizing a monomorphic epitope on HLA class I molecules. (b) Binding of the donor-specific anti-HLA antibodies out of the recipient's serum to the HLA molecules of the donor. (c) Binding of alkaline phosphatase conjugated secondary antibodies to the recipient's bound donor-specific anti-HLA class I antibodies and subsequent color reaction. The original protocol was modified by substituting the human IgG-specific antibody by a human IgG/M/A-specific secondary antibody. (d) Lysate control using an alkaline phosphatase conjugated monoclonal detection antibody directed against a second monomorphic epitope to confirm the immobilization of a sufficient amount of HLA molecules by the solid-phase-bound capture antibody. The AMS-ELISA variant for the identification of donor-specific antibodies directed against HLA class II molecules is correspondingly designed.

A 42-year-old prospective female lung recipient suffering from cystic fibrosis was HLA-phenotyped and genotyped for HLA class I antigens HLA-A11,26; B27,62 (Bw4,6); and Cw2,3 and genotyped for HLA class II antigens HLA-DR13,15; DR51,52; and DQ6 to be registered on the waiting list for lung allografting. Due to immunizations of unknown origin the patient had already been immunized for some HLA antigens when entering the waiting list in 08/2012. Anti-HLA antibody screening for the detection of anti-HLA class I and anti-HLA class II antibodies was routinely performed as a first approach using the QuikScreen and B-Screen assays (GTI Diagnostics, Waukesha, USA). As the anti-HLA class I screening assay was positive antibody specification/identification was performed to define anti-HLA class I and antibody specificities. The first specifications using Luminex-based assays (Lifecodes/Immucor, Stamford, USA) at the level of Single Donor (single ID) resolution exhibited panel-reactive antibodies (PRA%) of 54% against HLA class I and of 0% against HLA class II molecules, respectively, thus confirming a certain level of immunization against HLA class I molecules and determining the anti-HLA antibody specificities as anti-HLA-A9 (23,24), anti-HLA-B7, anti-HLA-B27, anti-HLA-B40 (60,61), anti-HLA-B47, and anti-HLA-B81 (Table 1(a)). After about six months on the waiting list (02/2013) a lung allograft was offered to the patient and transplanted. It was typed HLA-A3; B7,38 (Bw4,6); and Cw7,12 for class I and DR13; DR52; and DQ6 for class II. Thus, the resulting mismatch scheme of the graft covering only the A-B-DR antigens regarded as most important was determined as 1-2-0 (A-B-DR MM). Due to the incompatibility of the Cw antigens two theoretical HLA targets existed whereas the HLA class II antigen DQ6 was present on the patient's as well as on the allograft's cells theoretically representing no additional antigen. Consequently the only specificity of the patient leading to a positive virtual crossmatch result was due to the anti-HLA-B7 antibodies identifiable in the patient's serum and theoretically directed against the HLA-B7 antigen expressed on the allograft's cells. It is noteworthy in this context that due to the guidelines of the Eurotransplant Foundation and of the German Federal Medical Association a pretransplant CDC-CM which is strictly mandatory prior to kidney allografting is generally not required as a prerequisite of lung transplantations. Unfortunately, the donor's antigen HLA-B7 based on preformed antibodies of the recipient (leading to a positive virtual crossmatch) was overlooked for representing so-called “unacceptable antigens” and allografting was performed. After five months a clinically apparent rejection episode was observed (07/2013) and our laboratory was asked for analyzing anti-HLA antibodies as possible cause. In best accordance with the antibody specifications performed in order to fulfill the criteria for the waiting list entry all of the above mentioned anti-HLA antibodies were clearly identifiable using Luminex analysis for a second time exhibiting a very similar PRA of 60% (Table 1). Since a deep-frozen (−28°C) detergent lysate derived from spleen leukocytes from that lung donor was stored in our laboratory the idea arose to perform crossmatching using this material available for us because the typing of that given donor had accidentally been performed in our laboratory about five months ago. The residual cell pellet had originally been stored for the purpose of DNA preparation. As no vital cells existed after thawing any vitality assay such as CDC-based crossmatching was a priori excluded. Thus, the alternative procedure of ELISA-based crossmatching using the AMS-class I/II ELISA (GTI, Waukesha, USA) was used by us which after its discontinuation by the manufacturer in 2013 was replaced by the AbCross HLA class I/II ELISA (Bio-Rad Medical Diagnostics, Dreieich, Germany) completely modified in our laboratory and resulting in a workflow which was nearly identical to the former AMS-ELISA. Both assays, however, allow the direct detection of DSA by immobilizing solubilized HLA molecules from a donor's cells/tissues onto which, in a consecutive step, only donor-specific but not anti-HLA antibodies in general bind. The principle of work is demonstrated in the flow scheme (Figure 1). Wells of ELISA strips (GTI) or Terasaki-Microtest plates (Bio-Rad), respectively, precoated with monoclonal capture antibodies were filled with detergent lysate of donor cells/tissues including HLA molecules. The capture antibodies are directed against monomorphic epitopes available on all HLA class I or class II molecules, respectively (Figure 1(a)). After this first incubation the wells were washed and incubated with the recipients' sera. If these sera contain DSA to be detected in this assay these represent detection antibodies in this ELISA recognizing the immobilized HLA molecules of a given donor (Figure 1(b)). After additional washing steps the wells were incubated with alkaline phosphatase (AMS-ELISA) or peroxidase (AbCross-ELISA) conjugated secondary anti-human IgG antibodies to recognize the immobilized primary donor-specific (detection) antibodies (Figure 1(c)), a step which could easily be modified using secondary anti-human IgG/M/A antibodies to identify other primary human isotypes. In order to validate the data the following controls were always performed: (i) the lysate controls (LCRI/II) consist of a second monoclonal antibody for the detection of immobilized HLA class I or class II molecules, respectively, by recognizing a second monomorphic epitope on the HLA molecules (HLA class I and class II/AMS-ELISA, HLA class II/AbCross-ELISA) or the beta 2 microglobulin (HLA class I/AbCross-ELISA). These positive controls provide evidence that a sufficient amount of the donors' HLA molecules was immobilized to get a signal. (ii) The negative controls of both assays use an irrelevant human serum which is negative for anti-HLA antibodies. The value of the serum under investigation has to exceed twofold the background value of the negative serum to be classified as positive. The serum taken at the date of the rejection episode (07/2013) was investigated for DSA against HLA molecules of the donor and clearly defined as positive for anti-HLA class I DSA at dilutions of 1 : 3 and 1 : 6 (Table 1). In contrast no DSA against HLA class II molecules were demonstrable (Table 1). Thus, this approach of using a five-month-old deep-frozen leukocyte lysate resulted in a successful diagnostic crossmatch using stored donor material to perform a* de facto* crossmatch in order to demonstrate the existence of DSA not only on the basis of virtual crossmatching. However, the donor's cell pellet had been too small to get sufficient detergent lysate for a continuing series of monitoring DSA. As a consequence only one follow-up analysis using the AMS-ELISA was performed after three apheresis cycles (08/2013) which clearly showed that this therapeutic approach did not exhibit any decrease in DSA in complete accordance with the detection of other anti-HLA class I antibodies still exhibiting a PRA of 58%. DSA were still detectable at 1 : 3 and 1 : 6 dilutions of the patient's serum and the spectrum of anti-HLA class I antibodies was completely the same as in the two previous Luminex-based analyses at the Single Donor (ID) level (Table 1). Further antibody-reducing therapeutic steps were monitored only through the use of Luminex analyses (not shown).

### 2.2. Case 2: Investigation of a Rejection Episode Using Donor's Material Stored for More Than Four Years to Clear That Anti-HLA Antibodies Are Most Probably Not Involved

In the second report a 48-year-old male patient was phenotyped and genotyped for HLA class I antigens HLA-A1,11; B8,35 (Bw6); and Cw4,7. For HLA class II antigens he was typed HLA DR1,7; DR53; and DQ5,3(9). In August 2010, that is, after only two months on the waiting list, the patient received a heart allograft from a donor typed HLA-A2; B7,51 (Bw4,6); and Cw7 for HLA class I and HLA-DR11,15; DR51,52; and DQ6,3(7) for HLA class II molecules. Since the donor was homozygous for HLA-A type A2 the resulting HLA A-B-DR mismatch scheme was 1-2-2. According to an agreement with the respective heart transplant center all heart recipients were retrospectively tested for DSA against the graft about two days after the transplantation when the material reached our tissue typing laboratory. Generally the AMS-ELISA (since 09/2013 the modified AbCross-ELISA) was used to detect DSA as the splenic material provided for this purpose contained only cells of insufficient vitality to be investigated using the CDC-CM. The analyses using a serum sample of the patient taken at the date of the transplantation but performed retrospectively, that is, two to three days afterwards, did exhibit neither DSA (AMS-ELISA) nor anti-HLA antibodies in general (GTI Screen ELISA and Luminex Single Donor analysis) (Table 2). Aliquots of the residual spleen-derived leukocytes' detergent lysate were deep-frozen (−30°C). The graft function was okay without any complications for a period of more than four years. Annually a routine check for the detectability of anti-HLA antibodies was performed generally using the GTI Screen ELISA for the detection of anti-classes I and II antibodies and additionally using Luminex (Single Donor) analyses in the years 2012 and 2014. Over four years all these assays never led to the conclusion that anti-HLA antibodies may be involved in impairing graft function and graft survival (Table 2). Thus, unexpectedly a clinically proven rejection episode was diagnosed in March 2015 leading to an immediate analysis of the patient's serum sample in our laboratory. As is visible in Table 2 no analyses for anti-HLA antibodies in general [GTI anti-HLA class I/II Screen ELISA, Luminex Single Donor anti-HLA class I/II assay, Lambda Antigen Tray anti-HLA Single Antigen ELISA (LAT 1HD), and Lambda Antigen Tray anti-HLA class I/II Single Donor ELISA (LAT12/88)] detected anti-HLA antibodies of any specificity leading to the conclusion of a negative virtual crossmatch. This result, however, was strongly supported by the outcome of a* de facto* crossmatch which used the residual splenic leukocytes' lysate deep-frozen for four years and seven months as donor material (Table 2). Also the modified AbCross-ELISA did not detect donor-specific anti-HLA class I and class II antibodies. Because strong reactions of both positive controls (LCRI/II) were demonstrable (Figure 1(d)) there was no doubt about the validity of the negative reaction using the patient's serum. Thus, evidence was provided that sufficient numbers of class I and class II HLA molecules were still available and had not been degraded. A sufficient number of HLA molecules could be immobilized to result in an adequate signal if DSA were part of the patient's serum although the donor's cell lysate had been stored for about 4.5 years. This investigation may lead to new diagnostic conceptions using long time-stored donor material to define and monitor humoral rejection episodes after solid organ allografting characterized by an involvement of DSA.

### 2.3. Case 3: Identification of Donor-Specific and Additionally Allele-Specific Antibodies Not Definable by Virtual Crossmatching at the Low (Two-Digit) Resolution Level

Four years ago a case was investigated in our laboratory dealing with a very special situation when unforeseeable allele-specific antibodies led to the loss of a kidney allograft. These had arisen although the patient had received a fully matched postmortem kidney as defined by virtual crossmatching at the level of two-digit resolution. The 10-year-old male patient with end stage renal insufficiency was HLA-typed A3,25; B8,18 (Bw6); Cw7,12; DR15,17; DR51,52; and DQ6 (Table 3(a)). In 1998 he received a graft with no HLA mismatch. Due to the donor's homozygosities in DR and DQ phenotypes no rejection targets were offered leading to the most favorable HLA A-B-DR MM scheme 0-0-0 (Table 3(a)). As required by the guidelines for kidney allografting in those days and as expected on the basis of perfect HLA matching the pretransplant CDC-based crossmatch performed in 1998 was negative for PBL, T-cells, and B-cells, as a matter of course leading to the transplantation. However, unexpectedly this allograft lost its function after eight years leading to the reentry of the patient onto the waiting list in the year 2006. Using the HLA classes I and II antibody screening ELISA (GTI Diagnostics) and afterwards Luminex Single Donor as well as LAT 1HD Single Antigen ELISA analyses anti-HLA-A25, anti-HLA-A26, anti-HLA-A34, and anti-HLA-A66 antibodies were clearly identifiable whereas anti-HLA class II antibodies were excluded (Table 3(a)). All of these so-called split antigens belong to the common (broad) antigen HLA-A10 strongly suggesting that antibodies against this common epitope had been generated. Thus, the situation in 2006 was that virtual crossmatching at the two-digit level did not clear the situation as anti-HLA-A25 antibodies theoretically represented anti-HLA autoantibodies. This, however, is an ultrarare immunological phenomenon. In most cases the pseudoidentification of anti-HLA autoantibodies results from artificially positive outcomes of the CDC-CM (in this case the CDC-based autocrossmatch) highly susceptible to various artefacts and disruptive factors which in many cases remain unknown due to insufficient consecutive diagnostic follow-up analyses [12, 15, 16, 22]. In contrast real anti-self-HLA class II antibodies have recently been detected in cases of autoimmune hepatitis [23]. When reentering the waiting list for kidney allografting the meanwhile 18-year-old patient, however, did not exhibit a positive CDC-based autocrossmatch nor did he suffer from any autoimmune disease. Thus, anti-HLA autoantibodies were not at all probable right from the beginning of the patient's antibody specification. Nevertheless a kidney offer in the year 2009 had to be refused due to a positive pretransplant CDC-based crossmatch (Table 3(a)) although virtually no antibodies were detectable against this potential graft. The conclusion that indeed DSA had been the reason for the allograft loss and the refusal of the kidney offer in 2009 was finally based on high resolution genotyping of the patient which exhibited the very rare allele HLA-A^*∗*^25:14 and not, as expected, the most frequent allele HLA-A^*∗*^25:01. Of course no material of the donor from 1998 was available after the patient's graft loss and his reentry onto the waiting list. However, in view of the antibody specification analyses which exhibited only anti-HLA-A10 (anti-A25) antibodies it is highly probable that the immune response led to DSA against the phenotype of the donor's frequent allele HLA-A^*∗*^25:01. Furthermore, the AMS-ELISA used as an HLA-specific autocrossmatch assay in the year 2009 was negative as well. To retrospectively support this hypothesis of allele-specific DSA the AMS-ELISA was used to detect the respective patient's DSA against three lysates of stored and selected donor leukocyte lysates exhibiting the HLA-A25 phenotype derived from the frequent HLA-A^*∗*^25:01 allele (Table 3(b)). All runs of the AMS-crossmatch ELISA using three serum samples of the patient (06/2009, 07/2011, and 12/2012) resulted in unequivocally positive signals exhibiting anti-HLA class I DSA against these “donors” whereas DSA against HLA class II molecules were not detectable (Table 3(b)). If adequate material of the donor in 1998 had been available it is easy to conclude that the ELISA-based crossmatch assay could have been used immediately after the graft loss to investigate the patient's serum for DSA against stored material of the donor of that kidney allografted in 1998. Thus, allele-specific DSA as the reason for the rejection of a virtually HLA-identical graft would most probably have been detected earlier and with higher validity/plausibility by* de facto* crossmatching than by unmeaning two-digit virtual crossmatching alone. This only led to the right conclusions with a long way round gone by high resolution (four-digit) typing.

## 3. Discussion and Conclusions

Based on the three exemplary cases it was the aim of the current report to present data on the possibility to reliably use deep-frozen cell pellet's detergent lysate of a given donor's material. The reported cases represent the first approach to reliably and routinely use deceased donor's material in order to monitor upcoming donor-specific anti-HLA antibodies accompanying a rejection episode by* de facto* crossmatching. Thus, this report to our best knowledge describes the first approach suitable for any laboratory's routine task to reliably monitor a humoral alloreactive anti-HLA immune response through the use of a deceased donor's stored material and not solely based on virtual crossmatching as a theoretical approach.

An increasing number of drawbacks of the CDC-CM procedure, established as the prototype of crossmatching in the late sixties, have been reported in the last 10 to 15 years [4, 5, 11, 12, 14–20] mainly in the context of this assay's insufficiency in leading to valid results under certain prerequisites. These publications clearly demonstrate that the CDC-CM under several surrounding circumstances has hardly the capacity to result in a reliable and, in the whole context of immunological diagnostics, plausible identification of DSA. The discrepancies reported are due to the fact that the CDC-CM as a vitality assay which depends on the activation of added complement components is highly susceptible to artificial factors which do not represent DSA but also lead to an activation of the complement system. Consequently these artefacts falsify this assay's outcome by simulating DSA-mediated positive reactions. Pharmaceutical treatment, especially the use of therapeutic monoclonal antibodies of complement-activating isotypes such as Rituximab and Basiliximab both of which belong to the IgG1 isotype, is noteworthy in this context [14–17]. Furthermore, autoimmune diseases especially those of the immune complex type III such as Systemic Lupus Erythematosus and Rheumatoid Arthritis just as much lead to false positive results of the CDC-CM [4, 16, 18–20].

Regardless of these artefact-mediated false positive outcomes which have increasingly been discussed during the last years to severely hamper the allocation of deceased donors' kidneys to certain groups of patients on the kidney waiting list it was the aim of the current report to point to the methodic aspect of using stored donor's material to demonstrate the upcoming DSA against a given deceased organ donor. Generally in these cases DSA are solely identified by virtual crossmatching, that is, by the comparison of antibody specificities of a given recipient with the HLA antigens of the corresponding donor. Limitations of virtual crossmatching based on HLA typing at the two-digit level, where only this is required for allografting solid organs, are shown using the example of case 3. ELISA-based crossmatching first provides a diagnostic tool to overcome this limitation through the use of a given donor's retained sample which consists of nonvital, that is, deep-frozen, and, in the long term, storable material. To the best of our knowledge the only approach to arrange a* de facto* crossmatch using stored material was historically performed 18 years ago in the context of corneal grafting. As donors' postmortem vital material, retinal pigment epithelial cells taken from explanted eyes were used. For this purpose the cells which were isolated and stored in liquid nitrogen had to be recultured and stimulated with IFN-*γ* to upregulate the expression of HLA molecules for a subsequent flow cytometry-based crossmatch analysis [24]. However, this historical approach mandatorily using vital cells must be regarded as technically very challenging, time-consuming, and expensive. Thus, as a matter of fact it is inappropriate for the routine task of any tissue typing laboratory in contrast to the ELISA-based procedure presented here. The most prominent advantage of ELISA-based crossmatching is that it does not require vital lymphocytes or other vital cells in general. Furthermore, in terms of technical and terminable practicability of about 3.5 hours it is easily implementable in any laboratory without expensive technical equipment.

The idea to perform* de facto* instead of virtual crossmatching is strongly supported by an increasing number of so-called allele-specific antibodies described in literature [25–27]. These antibodies are directed only against one allele or a limited number of alleles of an HLA phenotype defined by the two-digit level of resolution which as a matter of fact represents a whole group of alleles and may be characterized by various epitopes on different alleles. Therefore, the typing results performed at the level of two-digit typing of a given donor do not always plausibly allow the virtual identification of DSA. These antibodies may virtually appear as autoantibodies [22, 25–27] although directed against another allele of the same HLA antigen as defined at the two-digit level of resolution. Furthermore, DSA defined by virtual crossmatching are generally not detectable if they are directed against phenotypes of rare alleles not immobilized as antigens in the various specification/identification assays commercially available. However, these antibodies are necessarily detectable if material of the real donor (i.e., material indeed carrying the donor's rare HLA molecules) is used.

Of course a sufficient amount of donor-derived material is required to be stored for a more systematic approach, that is, in order to store material sufficient for several future attempts of ELISA-based crossmatching including all donors. This has not been done for the initial exemplary cases presented here as it was not the historical aim to store donor material in order to detect or exclude emerging anti-HLA DSA. However, if splenic tissue is provided and this is generally available from nearly all of the postmortem donors, there is no problem to systematically and adequately store sufficient material and to provide something like a tissue bank comprising all donors for this special application. The systematic approach of providing sufficient donor material in order to enable at least five consecutive crossmatch-ELISA-based analyses using double preparations of the respective recipient's serum at two dilution steps (1 : 3 and 1 : 6) has been followed in our laboratory since November 2014 for all lung and heart recipients.

Taken together the cases presented in the current reports clearly demonstrate the benefit by implementing ELISA-based crossmatching as an alternative methodic approach first allowing the usage of stored donors' materials in order to monitor an upcoming donor-specific anti-HLA immune response.

## Figures and Tables

**Table 1:** Results of Luminex-based anti-HLA antibody specification analyses and corresponding outcomes of the AMS-crossmatch ELISA for the 42-year-old lung recipient highlighting an involvement of anti-HLA DSA (anti-HLA-B7).

Serum sample	Luminex (Single Donor/ID) Ab specificities	PRA (%)	AMS-ELISA-CM	
			Class I	Class II
08/2012^*∗*^	Class I: anti-A9 (23, 24), anti**-B7,**	54%	n.d.	n.d.
	anti-B27, anti-B40 (60, 61), anti-B47, and anti-B81			
	Class II: neg.	0%		

07/2013^#^	Class I: anti-A9 (23, 24), anti**-B7,**	60%	**pos.**	neg.
	anti-B27, anti-B40 (60, 61), anti-B47, and anti-B81		**(1** : **6)**	
	Class II: neg.	0%		

08/2013^§^	Class I: anti-A9 (23, 24), anti**-B7,**	58%	**pos.**	neg.
	anti-B27, anti-B40 (60, 61), anti-B47, and anti-B81		**(1** : **6)**	
	Class II: neg.	0%		

n.d.: not done; neg.: negative; pos.: positive; ^*∗*^antibody specification as prerequisite for entering the waiting list; ^#^antibody analyses at the date of the rejection episode; ^§^antibody analyses after three apheresis cycles; bold lettering: donor-specific antibodies as detected by virtual (Luminex) or *de facto* (AMS-ELISA) crossmatching at the highest dilution (parentheses).

**Table 2:** Results of different antibody detection and specification analyses in comparison with crossmatch-ELISA outcomes all of which exclude an involvement of anti-HLA DSA in a rejection episode of the 48-year-old heart recipient with high probability.

Serum sample	GTI screening	Luminex (SD)	AMS-ELISA-CM
	Class I/II ELISA	Class I/class II	Class I/class II
06/2010^&^	neg./neg.	n.d./n.d.	n.d./n.d.
08/2010^$^	neg./neg.	neg./neg. (PRA = 0%)	neg./neg.
07/2011^*∗*^	neg./neg.	n.d./n.d.	n.d./n.d.
08/2012^*∗*^	neg./neg.	neg./neg. (PRA = 0%)	n.d./n.d.
08/2013^*∗*^	neg./neg.	n.d./n.d.	n.d./n.d.
11/2014^*∗*^	neg./neg.	neg./neg. (PRA = 0%)	n.d./n.d.
03/2015^#^	neg./neg.	neg./neg. (PRA = 0%)	neg./neg. (AbCr.)

For 03/2015 additionally:			
LAT 1HD (anti-HLA class I Single Antigen ELISA): negative			
LAT 1288 (anti-HLA class I/II Single Donor ELISA): negative			

n.d.: not done; neg.: negative; pos.: positive; PRA%: panel-reactive antibodies %; SD: Single Donor/PRA resolution; ^&^analyses for entering the waiting list; ^$^analyses at the date of the transplantation; ^*∗*^routinely performed posttransplantation analyses; ^#^analyses at the date of the clinically proven rejection episode; (AbCr.): after its discontinuation in 2013 the AMS-ELISA was replaced by the highly modified AbCross-ELISA.

**(a):** 

Patient's typing	Donor's typing (1998)^§^	Typing of the refused kidney offer (2009)^#^
A3,25 (10)	A3,25 (10)	A3,25 (10)
A^*∗*^03 : 01	(high res. not done)	A^*∗*^03 : 01
**A** ^**∗**^25 : 14		A^*∗*^25 : 01
B8,18 (Bw6)	B8,18 (Bw6)	B18,49 (Bw4,6)
Cw7,12	Cw7,12	Cw7,12
DR15,17	DR15,15	DR4,14
DR51,52	DR51,51	DR52,53
DQ2,6	DQ6,6	DQ3 (8),5

*Patient's identified anti-HLA antibodies*: anti-A25 (10); other HLA-A10 specifications: anti-A26 (10), anti-A34 (10), and anti-A66 (10).		

^§^Crossmatch results using the patient's pretransplant serum from 1998: negative CDC-based pre-TX crossmatch in 1998 with PBL, T-cell, and B-cell.

^#^Crossmatch results using the patient's pretransplant serum from 06/2009: positive CDC-based pre-TX crossmatch in 2009 with PBL, T-cell, and B-cell.

**(b):** 

Patient's serum sample	AbCross-ELISA-CM results against selected virtual donors		
	B.H.	K.P.	T.H.
	class I/II	class I/II	class I/II
06/2009	pos./neg.	pos./neg.	pos./neg.
07/2011	pos./neg.	pos./neg.	pos./neg.
12/2012	pos./neg.	pos./neg.	pos./neg.

Virtual donors' HLA-A high res. typing results: B.H.: HLA-A^*∗*^24:02, ^*∗*^25:01, K.P.: HLA-A^*∗*^25:01, ^*∗*^26:01, T.H.: HLA-A^*∗*^02:01, ^*∗*^25:01.			

## Abbreviations

AMS: Antibody Monitoring System
CDC: Complement-dependent cytotoxicity
CM: Crossmatch
DSA: Donor-specific antibodies
ELISA: Enzyme-linked immunosorbent assay
FACS-CM: Flow cytometric crossmatch
HLA: Human leukocyte antigen
mAb: Monoclonal antibody
PRA: Panel-reactive antibodies.
